# Genital Self-Mutilation in a Patient with Psychosis: A Case of Klingsor Syndrome

**DOI:** 10.1192/j.eurpsy.2023.1310

**Published:** 2023-07-19

**Authors:** M. S. Yildirim, B. N. Guvendi Melenkis, S. C. Paltun

**Affiliations:** Psychiatry Department, Erenköy Training and Research Hospital for Psychiatry and Neurological Diseases, University of Health Sciences, Istanbul, Türkiye

## Abstract

**Introduction:**

Intentionally harming oneself with deliberate destruction of body parts without a suicidal purpose is defined as self-mutilative behavior. Genital Self-Mutilation is an extreme form of such action and usually seen as a result of an underlying psychiatric condition. Although schizophrenia spectrum disorders are the leading cause, substance use, personality disorders, and gender dysphoria may also result in GSM. Klingsor Syndrome, a rare clinical entity, was first described as GSM with religious delusions. Later, Schweitzer proposed expanding the term to include all psychotic disorders. (Veeder et al., Gen Hosp Psychiatry 2017;44:43-50)

**Objectives:**

The aim of this piece is to report our case of a patient with psychosis performing genital self-mutilation in order to promote proper diagnosis and management of patients with similar conditions.

**Methods:**

A 24-year-old male was brought to the psychiatric emergency unit after self-harming behavior causing numerous wounds throughout his face, trunk, and genital area. Penile and scrotal lacerations were prominent. The patient stated that he had inflicted these wounds upon command hallucinations. Examination also revealed disorganized speech, dysphoric mood, paranoid delusions. The wounds were healed and the patient was prescribed antibiotic medication. He was then admitted to the psychiatric ward. The patient’s first psychiatric visit was dating back to four years prior to his inpatient admission. However, symptoms of paranoid delusions and auditory hallucinations had been more severe for about a year. Throughout his outpatient appointments during this time, olanzapine and aripiprazole were tried and a partial response was elicited but the patient generally was non-compliant with the treatment. Cannabis use history was also significant. On admission, the patient was put on amisulpride 800 mg daily, gradually increased to 1200 mg. Valproic acid 1000 mg/day was also added to the treatment in order to control impulsive behaviors. Care of the genital wounds was provided as per the recommendations of the urology department. Near total improvement of the psychiatric symptoms were achieved during the hospitalization. The final diagnosis of Klingsor Syndrome was concluded as the patient was discharged.

**Results:**

The patient’s remission sustained during outpatient follow-ups. About a year after discharge, asymptomatic prolactinemia was detected and managed by reducing amisulpride dose and addition of aripiprazole 5 mg/day.

**Conclusions:**

GSM is a dramatic form of self-harm. The severity of psychotic illness of patients often facilitates the conduction of such behaviors. Appropriate antipsychotic treatment and effective care may prevent patients from inflicting severe damage to themselves. Also, In cases of GSM in patients with underlying psychiatric conditions, an interdisciplinary approach is required.

**Disclosure of Interest:**

None Declared

